# Early Cellular, Molecular, Morphological and Behavioral Changes in the Humanized Amyloid-Beta-Knock-In Mouse Model of Late-Onset Alzheimer’s Disease

**DOI:** 10.3390/cells11040733

**Published:** 2022-02-19

**Authors:** Sudhir Kshirsagar, Rainier Vladlen Alvir, Ashly Hindle, Subodh Kumar, Murali Vijayan, Jangampalli Adi Pradeepkiran, Arubala P. Reddy, Bhagavathi Ramasubramanian, P. Hemachandra Reddy

**Affiliations:** 1 Department of Internal Medicine, Texas Tech University Health Sciences Center, Lubbock, TX 79430, USA; sudhir.kshirsagar@ttuhsc.edu (S.K.); makeitrain425@yahoo.com (R.V.A.); ashly.hindle@ttuhsc.edu (A.H.); subodh.kumar@ttuhsc.edu (S.K.); murali.vijayan@ttuhsc.edu (M.V.); pkiran82@gmail.com (J.A.P.); b.ramasubramanian@ttuhsc.edu (B.R.); 2 Nutritional Sciences Department, College of Human Sciences, Texas Tech University, 1301 Akron Ave, Lubbock, TX 79409, USA; arubala.reddy@ttu.edu; 3 Neuroscience & Pharmacology Department, Texas Tech University Health Sciences Center, Lubbock, TX 79430, USA; 4 Neurology, Departments of School of Medicine, Texas Tech University Health Sciences Center, Lubbock, TX 79430, USA; 5 Public Health Department of Graduate School of Biomedical Sciences, Texas Tech University Health Sciences Center, Lubbock, TX 79430, USA; 6 Department of Speech, Language and Hearing Sciences, School Health Professions, Texas Tech University Health Sciences Center, Lubbock, TX 79430, USA

**Keywords:** amyloid beta, mitochondria, late-onset Alzheimer’s disease, dendritic spines

## Abstract

The purpose of our study is to investigate early cellular, molecular, morphological and behavioral changes in humanized amyloid-beta-knock-in (hAbKI) mice. Using seven-month-old homozygous hAbKI mice, we studied behavioral phenotype parameters, including spatial learning and memory (Morris Water Maze), locomotor activity (open field), working memory (Y-maze) and motor coordination (rotarod); mRNA abundance, protein levels, soluble amyloid-beta 40 and 42 levels and regional immunoreactivities of key markers of mitochondrial dynamics, mitochondrial biogenesis, synaptic health, mitophagy and autophagy; mitochondrial function and using transmission electron microscopy & Golgi–Cox staining, we assessed mitochondrial morphology and dendritic spines. Our extensive behavioral analysis revealed that seven-month-old hAbKI mice showed impairments in motor coordination, reduced locomotor and exploration activities, impairments in working memory and spatial learning and memory. Our mRNA and protein analyses revealed the increased expression of mitochondrial-fission genes and reduced expression of mitochondrial-fusion, mitochondrial-biogenesis, synaptic, autophagy and mitophagy genes in seven-month-old hAbKI mice. An immunofluorescence analysis revealed altered immunoreactivities and agreed with the immunoblot results. Transmission-electron-microscopy data revealed increased mitochondrial fragmentation and reduced mitochondrial length in both hippocampal and cortical tissues of seven-month-old hAbKI mice and mitochondrial function defective. A Golgi–Cox-staining analysis revealed reduced dendritic spines in both cerebral cortices and hippocampi of hAbKI mice. Soluble amyloid-beta (1–40 and 1–42) were detected in three-month-old hAbKI mice and progressively increased in seven-month-old mice. These observations suggest that the human amyloid-beta peptide is sufficient to cause behavioral, mitochondrial, synaptic and ultrastructural changes in seven-month-old hAbKI mice. Our study findings also suggest that hAbKI mice might serve as a model for preclinical studies of preventive therapies.

## 1. Introduction

Alzheimer’s disease (AD) is a progressive, neurodegenerative disorder and is the most common cause of dementia in older people [1,2] Currently, over 50 million people worldwide are living with AD-related dementia, and this number is expected to increase to 152 million by 2050 (Alzheimer Association 2021). In addition to dementia, AD is associated with the loss of synapses [3,4], synaptic dysfunction [5], mitochondrial structural and functional abnormalities [6,7,8], microRNA deregulation [9,10], inflammatory responses [11], neuronal loss, accumulation of amyloid-beta (Aβ) [12,13] and phosphorylated tau (p-tau) [14,15,16]. Despite the progress that has been made in better understanding AD pathogenesis, researchers have still not identified early detectable markers, drugs, or agents that can prevent AD or slow its progression. The causes of AD are largely still unknown, and no curative treatments exist to date [17,18,19].

AD occurs in two forms: early-onset familial (FAD) and late-onset sporadic (SAD). FAD is rare and clinical symptoms develop mostly between the ages of 30 and 50. FAD is caused by genetic mutations in amyloid-beta-precursor protein (APP), presenilin1 (PSEN1), and presenilin 2 (PSEN2), which lead to the overproduction of Aβ plaques [20,21,22,23]. In addition, the APOE4 genotype is a major risk factor for AD, and the age of disease onset is 65 years and beyond [24].

Increasing evidence suggests that multiple factors are involved in developing late-onset AD, including traumatic brain injury, cardiovascular, hypertension, kidney disease, diabetes/obesity, epigenetic conditions, environmental/occupational exposures and lifestyle conditions (unhealthy diet and physical inactivity) [19]. To understand the disease process, animal and cell models have been generated using genetic mutations in APP, PS1, PS2 loci, and APOE2, 3 and 4 alleles. The outcomes of these animal- and cell-model studies improved our basic understanding of the disease development, progression and pathogenesis.

Currently, there are over 170 AD animal models generated, in addition to invertebrate and non-mammalian models [25,26,27]. These transgenic mice that overexpress human AD genes involved in the production of amyloid-beta/neuritic plaques and phosphorylated tau/neurofibrillary tangles. Fly, worm, and fish models have been created with the similar approach of overexpressing human genes in order to generate plaques and tangles. Although these models have helped us to improve our understanding of AD progression and pathogenesis, their value is limited by a number of factors, including that most of the AD mice did not develop neurodegeneration or neuronal loss, and the models were focused mostly on familial AD and these models do not recapitulate the late-onset AD phenotypic behavior or AD pathologies. These factors limit the translatability of findings to AD patients.

To overcome the limitations of the available AD mouse models, Frank LaFerla’s group recently generated a late-onset AD mouse model using a humanized A-beta-knock-in (hAbKI) approach. These hA-beta-loxP-KI mice expressed endogenous mouse APP protein with a “humanized” Aβ peptide sequence. This model showed late-onset AD features representative of the vast majority of late-onset AD [28].

The key findings of this model include age-dependent impairments in cognition and synaptic plasticity, brain volumetric changes, inflammatory alterations, the appearance of periodic Schiff-acid granules (PAS) and changes in gene expression. In addition, the cre-mediated excision of exon 14 ablates hAβ expression, rescues cognition and reduces the formation of PAS granules. However, this published work did not study mitochondrial dynamics, biogenesis, autophagy, mitophagy or synaptic genes for mRNA abundance or protein levels. Further, this study did not investigate detailed behavioral phenotypes or ultrastructural changes in mitochondrial morphology and dendritic-spine density.

In the current study, using seven-month-old homozygous hAbKI mice and age-matched WT mice, we studied 1) behavioral-phenotype parameters, including spatial learning and memory (Morris Water Maze), locomotor activity (open field), working memory (Y-maze) and motor coordination (rotarod); 2) mRNA abundance and protein levels of mitochondrial fission (Drp1 and Fis1), mitochondrial fusion (Mfn1, Mfn2 and Opa1), mitochondrial biogenesis (PGC1a, Nrf1, Nrf2 and TFAM), synaptic health (synaptophysin, PSD95, Snap25, synapsin 1 & 2, synaptobrevins 1 & 2 and neurogranin), mitophagy (PINK1 and PARKIN) and autophagy (ATG5, Beclin, LC3A and LC3B); 3) we assessed cortical and hippocampal immunoreactivities of the above-mentioned proteins; 4) mitochondrial ultrastructural changes using transmission electron microscopy; and 5) dendritic-spine length and number using Golgi–Cox-staining analysis.

## 2. Materials and Methods

### 2.1. Humanized A-Beta-Knock-in (hAb-KI) Mice

We used the homozygous humanized knock-in mouse model (hAb-KI) because it has an entire mouse APP sequence with a human A-beta sequence [28]. The majority of human AD cases are late-onset AD (LOAD), yet most AD mouse models feature APP overexpression, which is more consistent with an early-AD-onset mouse model. The hAbKI AD mouse model is a recently established AD mouse model in which 3 amino acids within the A-beta region of the mouse APP sequence are “knocked in” to express human A-beta without changes to the 5′ and 3′UTR mouse sequences, that substantiates the use of hAb-KI mice for our proposed studies. Homozygotes are viable, fertile and live beyond 22 months. The hA-beta-loxP-KI allele is available on a C57BL6J/SJL congenic background (Stock No. 031050). Additionally, hAb-KI mice develop age-dependent changes in cognitive and synaptic plasticity, and an inflammatory response that mimics the late-onset progression seen in sporadic human AD cases. Breeding pairs were purchased from Jackson Labs, Bar Harbor, ME, USA.

Mice were bred and housed under standard 12 h light-dark cycle, with lights on at 7 AM in the Laboratory Animal Resource Center, Texas, Tech University Health Sciences Center, accredited by the Association for Assessment and Accreditation of Laboratory Animal Care International (AAALAC). All experimental protocols were approved by Texas Tech University Health Sciences Center, Institutional Animal Care and Use Committee (TTUHSC-IACUC).

We used 20 animals per group (meaning 20 hAbKI mice and 20 age-matched WT mice) for behavioral analysis and 5 animals for cell-biology (immunoblot, amyloid-beta levels) and molecular-biology (mRNA expression), 5 animals for immunofluorescence, 5 animals for transmission-electron-microscopy, and 5 animals for Golgi–Cox analyses per group.

### 2.2. Rotarod Test

A rotarod test was first used to measure the balance, coordination, and motor-planning of 7-month-old WT, hAbKI mice. To test differences in balance and motor coordination, said mice were placed on the mouse rotarod unit (Med Associates, Inc., St. Albans, VT, USA). The rotarod behavioral-assessment test was performed as previously described [29,30].

### 2.3. Open-Field Test

The open-field test was used to assay general locomotor-activity levels, willingness to explore, and anxiety-related behavior and tracked using the ANY-Maze^®^ software (Stoelting, Wood Dale, IL, USA). In a 40 cm square open field with video-tracking tools, 7-month-old WT, hAbKI mice were tracked for 5 min and under moderate lighting. The levels of general activity were measured by assessing the total distance traveled and average speed.

### 2.4. Y-Maze Test

Spatial learning and memory have been shown to be sensitive to hippocampal damage. The Y-maze test was used to measure spontaneous alternation for habituation and spatial working memory. Seven-month-old WT, HAbKI mice were allowed to freely explore all three arms of the Y-maze, and spontaneous alternation was calculated. Each mouse was placed in one of the arms and allowed one five-minute trial of free exploration of the three arms in the maze. The number of total arm choices and sequence of arm choices was recorded (ANY-Maze^®^, Stoelting, Wood Dale, IL, USA).

### 2.5. Morris-Water-Maze Test

Using the Morris-water-maze (MWM) test, we assessed spatial long-term memory and learning in hAbKI and WT mice [31,32]. The MWM test was conducted in a 120 cm-diameter, 50 cm-deep tank filled with opacified water (Utrecht Art Supplies, Cranbury, NJ, USA) kept at 25 ± 0.5 °C. A platform with a 9 cm diameter was submerged 1 cm under the water surface in a quadrant. The tank was divided into four quadrants NE, NW, SW, and SE. The platform was placed in the NW quadrant and remained at the same position during the whole experiment. Briefly, each group of animals was trained for four days in the MWM, with four trials per day. Each trial was allowed to run for one minute. If the animal did not find the platform within one minute, they were placed on the platform using the net for 3 s. After every trial, the animal was dried with a towel and placed into a holding cage. Animals were video tracked using ANY-Maze^®^ software (Stoelting, Wood Dale, IL, USA), and behavioral parameters, including average time to find the platform, distance traveled, and average speed were assessed [30,32].

### 2.6. qRT-PCR

Total RNA was isolated from cortical tissues from hAbKI and WT mice. We designed the oligonucleotide primers for the housekeeping genes β-actin, GAPDH, mitochondrial-structural genes, fission genes (Drp1, Fis1), fusion genes (MFN1, MFN2, Opa1), mitochondrial-biogenesis genes (PGC1a, Nrf1, Nrf2, TFAM) and synaptic genes (synaptophysin, PSD95, synapsins1-2, synaptobrevins1-2, neurogranin, and synaptopodin), autophagy/mitophagy genes (LC3B-I & II, LC3B, ATG5, Beclin1, PINK1, Parkin, and BCL2) using primer Express Software (Applied Biosystems, Carlsbad, CA, USA). The primer sequences and amplicon sizes are listed in Table 1. We performed RT-PCR using SYBR-Green chemistry-based quantitative real-time RT-PCR, as described by Kandimalla et al., 2016 [29].

### 2.7. Western-Blotting Analysis

Western-blot analysis was performed using cortical tissues from hAbKI and WT mice as described in lab publication by Kandimalla et al., 2016 [29]. We assessed mitochondrial dynamics, biogenesis, synaptic, autophagy and mitophagy protein. Beta-actin was used as a loading control in order to normalize the levels of mitochondrial biogenesis, dynamics, synaptic and mitophagy proteins. Details on antibodies and dilutions are given in Table 2.

### 2.8. Immunofluorescence Analysis

To determine the immunoreactivity and intensity of mitochondrial dynamics, biogenesis, synaptic, and autophagy/mitophagy proteins, we performed immunofluorescence analysis using brain sections of 7-month-old hAbKI and WT mice as described in our lab publications [30,32,33]. Details of antibody dilutions are given in Table 3. To quantify the immunoreactivities of antibodies, 10–15 photographs were taken at 4×, 10×, 20×, 40× and 100× magnification. Statistical significance was assessed by the intensities of red, green, or blue using NIH ImageJ software.

### 2.9. Dendritic Spine Analysis Using Golgi–cox Staining

Dendritic spines of neurons in the brains of 7-month-old WT, hAbKI mice were detected by Golgi–Cox staining, which was performed using the FD Rapid GolgiStain Kit (PK401, FD NeuroTechnologies, Columbia, MD, USA) as described earlier [30,32,33]. All procedures were performed under dark conditions. Mouse brain tissues were impregnated for 2 weeks and processed according to the manufacturer’s instructions. Briefly, dendrites within the CA1 subregion of the hippocampus and cerebral cortex were imaged using a 4×, 10×, 20×, and 100× objective using an AMG EVOS microscope (Thermo Fisher Scientific, Waltham, MA, USA) or an Olympus IX83 (Olympus Corporation, Tokyo, Japan). Approximately 20 neurons were randomly selected from each group and quantified with a double-blind, controlled design. ImageJ and Image-Pro Plus were used to evaluate the number of spines and the total dendritic length.

### 2.10. Transmission Electron Microscopy (TEM) of Brain Mitochondria

To determine the mitochondrial number and size, we performed transmission electron microscopy in hippocampal and cortical sections of 7-month-old WT, hAbKI mice using methods described in lab publications [30,32,33]. Analyses of mitochondria number and size in WT and hAbKI mouse brains were performed using Image J software. Briefly, mitochondria within a defined area of the field were identified and numbered by two independent, experienced researchers blinded from the details of each sample group. For measurement of mitochondrial number and size, ten random micrographs were taken from the hippocampus and cerebral cortex of each of the 3 pairs of WT and hAbKI (i.e., 30 micrographs each genotype) mice [30,32,33].

### 2.11. Measurement of Soluble Aβ Levels

Soluble Aβ levels were conducted using sandwich ELISA as described in Manczak et al. (2016) [34] and Manczak et al. (2018) [35]. A 96-well plate was used, following the manufacturer’s instructions. Each sample was run in duplicate. Protein concentrations of the homogenates were determined following the BSA method, and Aβ was expressed as pg Aβ/mg protein.

### 2.12. Mitochondrial Functional Assays

Using protein lysates from 7-month-old hAbKI and WT mice, we assessed mitochondrial function by measuring H_2_O_2_, lipid peroxidation, and ATP.

H_2_O_2_ production levels were measured using cell pellets as described in Reddy et al. (2018) [36]. Briefly, the reaction mixture contained mitochondrial proteins, Amplex Red reagents, horseradish peroxidase and a reaction buffer. The mixture was incubated at room temperature for 30 min, followed by spectrophotometer readings of fluorescence (570 nm). H_2_O_2_ production was determined using a standard curve equation expressed in nmol/μg mitochondrial protein.

Levels of 4-hydroxy-nonenol were measured using HNE-His ELISA Kit (Cell BioLabs, Inc., San Diego, CA, USA), as described in Reddy et al. 2018 [36]. Briefly, freshly prepared protein lysates were added to a 96-well protein-binding plate and incubated overnight at 4 °C. After washing 3 times, the anti-HNE-His antibody was added to the protein, which was then incubated for 2 h at room temperature. Next, the samples were incubated with a secondary antibody conjugated with peroxidase for 2 h at room temperature, followed by incubation with an enzyme substrate. Optical density was measured (at 450 nm) to quantify the level of HNE.

Mitochondrial ATP levels were measured using an ATP determination kit (Molecular Probes, Eugene, OR, USA), and ATP levels were measured in isolated mitochondria from cortical tissues from hAbKI and WT mice as described in Reddy et al. 2018 [36]. ATP levels were measured from mitochondrial pellets using a standard-curve method.

## 3. Results

### 3.1. Phenotypic Behavior

To determine the early behavioral impairments in hAbKI mice, we assessed locomotor activity/exploration abilities (open field), motor coordination (rotarod), working memory (Y-maze) and spatial learning and memory (Morris Water Maze) in seven-month-old hAbKI mice and age-matched WT mice.

#### 3.1.1. Rotarod Test

**Figure 1:**
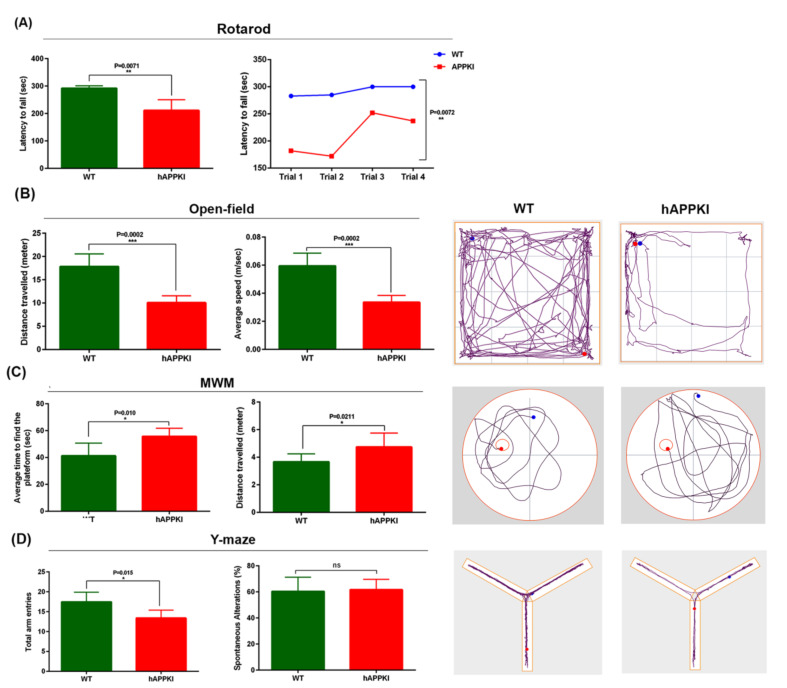
Cognitive behavior of seven-month-old hAbKI and WT mice. Phenotypic behavior is assessed using open field for locomotor activity/exploration abilities, rotarod for motor coordination, Y-maze for working memory and Morris Water Maze for spatial learning and memory in seven-month-old hAbKI mice and age-matched WT mice. (**A**) On an accelerating rotarod test, hAbKI mice did not stay longer compared to WT mice (*p* < 0.0071). (**B**) In open field, hAbKI mice showed reduced total distance traveled (*p* = 0.002) and average speed (*p* = 0.0002) compared to WT mice. (**C**) In the Morris-Water-Maze test, hAbKI mice showed an increased time to find the platform (*p* = 0.01), and increased distance traveled (*p* = 0.0211) compared with WT mice. (**D**) In the Y-Maze test the total number of arm entries was significantly reduced (*p* = 0.0180) and the percentage of spontaneous alternation between the arms of the Y-maze was significantly decreased (*p* = 0.015) for seven-month-old hAbKI compared age-matched WT mice. *, *p* = 0.01; **, *p* = 0.001; ***, *p* = 0.0001; ns, not significant.

In the accelerating rotarod test, hAbKI mice exhibited a reduced latency to fall compared to WT mice (*p* < 0.0071, Figure 1A), indicating that seven-month-old hAbKI mice exhibited motor deficits.

Open-field test: Compared to seven-month-old WT mice, hAbKI mice showed reduced total distance traveled (*p* = 0.0002) and reduced average speed (*p* = 0.0002) (Figure 1B). These observations indicate that locomotor activity is reduced in hAbKI mice.

#### 3.1.2. Morris Water Maze Test

As shown in Figure 1C, hAbKI mice showed an increased time to find the platform (*p* = 0.010, Figure 1C), and increased distance traveled (*p* = 0.0211, Figure 1C) compared with WT mice. These observations suggest that hAbKI mice exhibit spatial-memory impairments.

#### 3.1.3. Y-Maze Test

The total number of arm entries were significantly reduced (*p* = 0.0150) in seven-month-old hAbKI compared age-matched WT mice (Figure 1D). However, the percentage of spontaneous alternations did not change between WT and hAbKI mice. These results suggest that the spatial working memory was reduced for hAbKI mice.

### 3.2. Gene-Expression Differences between hAbKI Mice and WT Mice

#### 3.2.1. Mitochondrial-Dynamic Genes

Significantly increased levels of mRNA expression of the fission genes Drp1 (by 1.8-fold) and Fis1 (by 1.69-fold) were present in seven-month-old hAbKI mice relative to the age-matched WT mice (Table 4). On the other hand, the levels of mRNA expression of the mitochondrial-fusion genes Mfn1 (by 2.0-fold), Mfn2 (by 1.33-fold) and Opa1 (by 1.38-fold) were reduced in hAbKI mice relative to the age-matched WT mice. These findings indicate that human Aβ peptide increases fission activity and reduces fusion activity in seven-month-old hAbKI mice.

To understand the effects of humanized Aβ in mice on mitochondrial dynamics, biogenesis, synaptic, autophagy and mitophagy genes, we performed real-time qRT-PCR on cortical and hippocampal tissues from seven-month-old hAbKI mice and age-matched WT mice.

#### 3.2.2. Synaptic Genes

Decreased mRNA expression levels were found for synaptophysin (2.04-fold), PSD95 (1.1-fold), SNAP25 (2.27-fold), synapsin 1 (1.4-fold), synapsin 2 (5.0-fold), synaptobrevin 1 (1.26-fold), synaptobrevin 2 (2.4-fold) and neurogranin (1.54-fold) in seven-month-old hAbKI mice relative to the age-matched WT mice. These observations indicate that human Aβ peptide reduces synaptic activities (Table 4).

#### 3.2.3. Biogenesis Genes

Significantly reduced mRNA levels were found in PGC1α (1.7-fold), NRF1 (1.4-fold), NRF2 (1.36-fold) and TFAM (2.0-fold) in seven-month-old hAbKI mice relative to the age-matched WT mice, suggesting that human Aβ decrease mitochondrial biogenesis (Table 4).

#### 3.2.4. Mitophagy Genes

Significantly reduced mRNA levels were found in the mitophagy genes PINK1 (2.0-fold) and PARKIN (1.16-fold) in seven-month-old hAbKI mice relative to the Table 4). Significantly reduced mRNA expression of the autophagy genes ATG5 (1.6-fold), BCL2 (1.25-fold), and LC3B (1.33-fold) was found in seven-month-old hAbKI mice (Table 4).

### 3.3. Immunoblotting Analysis

To assess the effects of human Aβ peptide on mitochondrial-, synaptic-, autophagy-, mitophagy- and inflammation-protein levels, we performed an immunoblotting analysis on protein lysates prepared from seven-month-old hAbKI mice and age-matched WT mice.

#### 3.3.1. Mitochondrial-Dynamic Proteins

**Figure 2:**
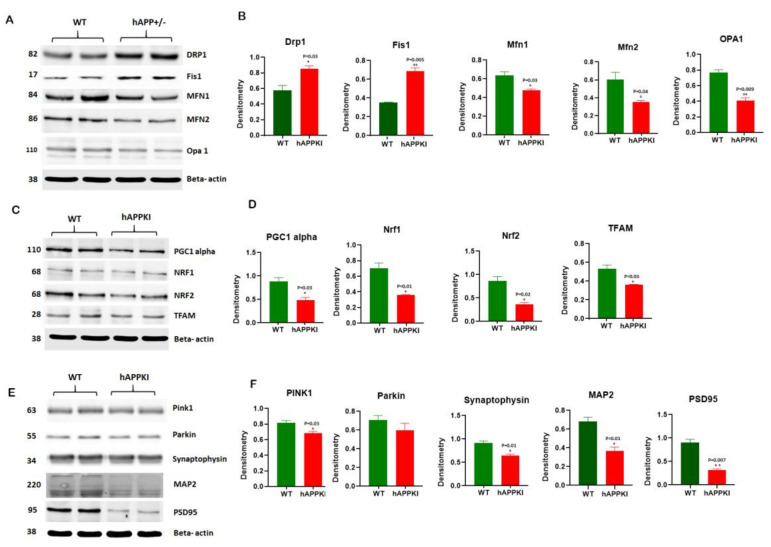
Immunoblotting analysis. Mitochondrial-dynamic, biogenesis, mitophagy, synaptic proteins and dendritic proteins MAP2 were assessed using lysates prepared from post-mortem brains of seven-month-old WT and hAbKI mice. (**A**) Representative immunoblots for WT and hAbKI mice. (**B**) Quantitative-densitometry analysis for mitochondrial-fission genes Drp1 and Fis1 and fusion proteins, which were significantly increased in the hAbKI mice compared to the WT mice. (**C**) Representative immunoblots for mitochondrial-biogenesis proteins in WT and hAbKI mice. (**D**) Quantitative-densitometry analysis of mitochondrial-biogenesis proteins PGC1α, NRF1, NRF2 and TFAM. PGC1α, NRF1, NRF2 and TFAM were significantly reduced in the hAbKI mice compared to the WT mice. (**E**) Representative immunoblots for untreated and WT mice and hAbKI mice. (**F**) Quantitative-densitometry analysis of PINK1, Parkin, synaptophysin, PSD95 and MAP2, which shows significant reduction in the hAbKI mice compared to the WT mice. *, *p* = 0.01; **, *p* = 0.001.

We found increased levels of the fission proteins Drp1 (*p* = 0.03) and Fis1 (*p* = 0.005) in seven-month-old hAbKI mice relative to the age-matched WT mice. By contrast, the mitochondrial-fusion proteins Mfn1 (*p* = 0.03), Mfn2 (*p* = 0.04) and Opa1 (*p* = 0.009) were significantly decreased in seven-month-old hAbKI mice relative to WT mice (Figure 2A,B).

#### 3.3.2. Mitochondrial Biogenesis

As shown in Figure 2C,D, significantly decreased levels of biogenesis proteins PGC1α (*p* = 0.03), NRF1 (*p* = 0.01), NRF2 (*p* = 0.02) and TFAM (*p* = 0.03) were found in seven-month-old hAbKI mice and age-matched WT mice, indicating that mitochondrial biogenesis was reduced in seven-month-old hAbKI mice relative to WT mice.

#### 3.3.3. Dendritic and Synaptic Proteins

Significantly decreased levels of the synaptic proteins synaptophysin (*p* = 0.01), PSD95 (*p* = 0.02) and MAP2 (*p* = 0.04) were found in seven-month-old hAbKI mice relative to WT mice (Figure 2E,F).

#### 3.3.4. Mitophagy Proteins

As shown in Figure 2E,F, the mitophagy proteins PINK1 (*p* = 0.03) and Parkin (*p* = 0.01) were reduced in seven-month-old hAbKI mice relative to WT mice.

#### 3.3.5. Autophagy Proteins

**Figure 3:**
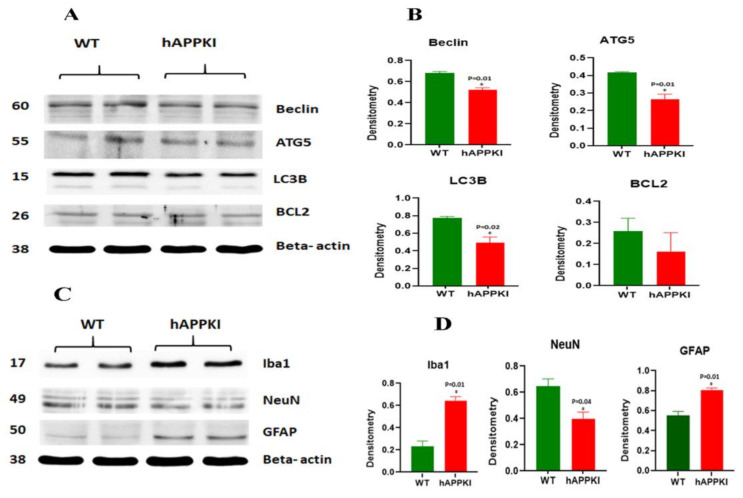
Immunoblotting analysis of autophagy proteins, NeuN, microglial marker Iba and astrocytic marker GFAP in protein lysates obtained from brains of seven-month-old WT and hAbKI. (**A**) Representative autophagy immunoblots for WT and hAbKI mice. (**B**) Quantitative-densitometry analysis for autophagy proteins ATG5, Beclin, BCL2, LC3B-I and LC3B-II showed they were significantly reduced in the APP mice compared to the WT (ATG5 *p* = 0.02; Beclin *p* = 0.01; LC3B-I *p* = 0.02, LC3B-II *p* = 0.02). **(C)** Representative immunoblots for microglia Iba, astrocytes GFAP and neuronal marker NeuN in WT and hAbKI mice. (**D**) Significantly reduced levels of the neuronal marker NeuN (*p* =0.04), and increased levels of microglial marker Iba (*p* =0.01) and astrocytic marker GFAP (*p* = 0.01) in -seven-month-old hAbKI mice relative to WT mice. *, *p* = 0.01.

Significantly decreased levels of the autophagy proteins ATG5 (*p* = 0.01), LC3BI (*p* = 0.02), LC3BII (*p* = 0.02) and Beclin (*p* = 0.01) were found in seven-month-old hAbKI mice relative to WT mice (Figure 3A,B).

#### 3.3.6. Inflammatory Response Proteins

As shown in Figure 3C,D, significantly reduced levels of the neuronal-marker protein NeuN (*p* = 0.04), and increased levels of the microglial marker Iba (*p* = 0.01) and the astrocytic marker GFAP (*p* = 0.01) were observed in seven-month-old hAbKI mice relative to WT mice.

### 3.4. Immunofluorescence Analysis

We performed an extensive immunofluorescence analysis of cortical and hippocampal brain sections for all proteins that were assessed by Western blot. The findings from our immunofluorescence analysis of mitochondrial-dynamic, biogenesis, mitophagy, autophagy and synaptic proteins were in agreement with our immunoblotting results.

**Figure 4:**
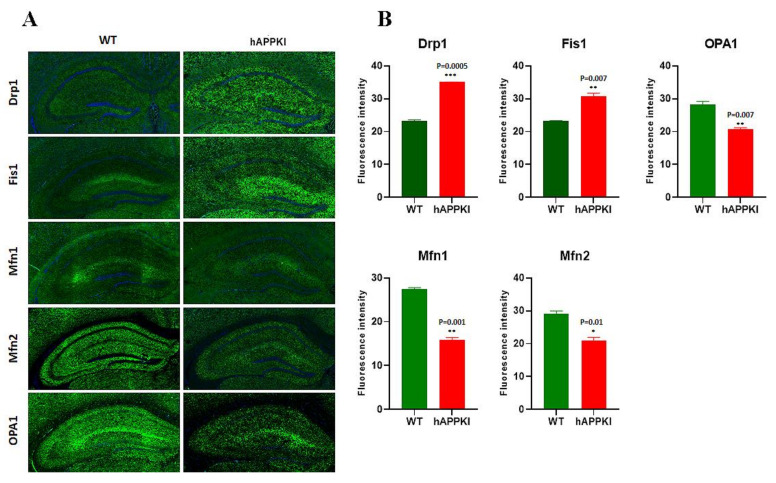
Immunofluorescence analysis of hippocampal mitochondrial-fission and mitochondrial-dynamic proteins in seven-month-old WT and hAbKI mice. (**A**) Immunofluorescence staining and quantitative-immunofluorescence analysis of WT and hAbKI mice. (**B**) Drp1 and Fis1 levels were significantly elevated (Drp1 *p* = 0.0005: Fis1 *p*= 0.007) in the hAbKI mice. *, *p* = 0.01; **, *p* = 0.001; ***, *p* = 0.0001.

**Figure 5:**
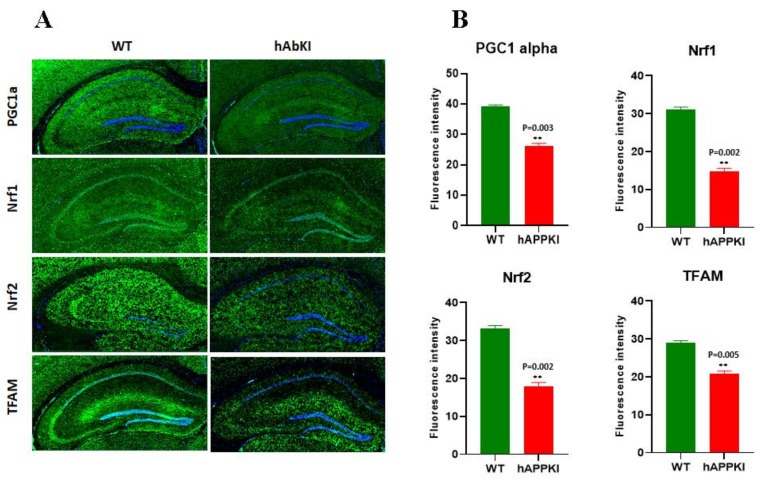
Immunofluorescence analysis of hippocampal mitochondrial-biogenesis proteins in seven-month-old WT and hAbKI mice. (**A**) Representative immunofluorescence images and (**B**) quantitative analysis of mitochondrial-biogenesis proteins, PGC1a, Nrf1, Nrf2 and TFAM in hAbKI mice. **, *p* = 0.001.

**Figure 6:**
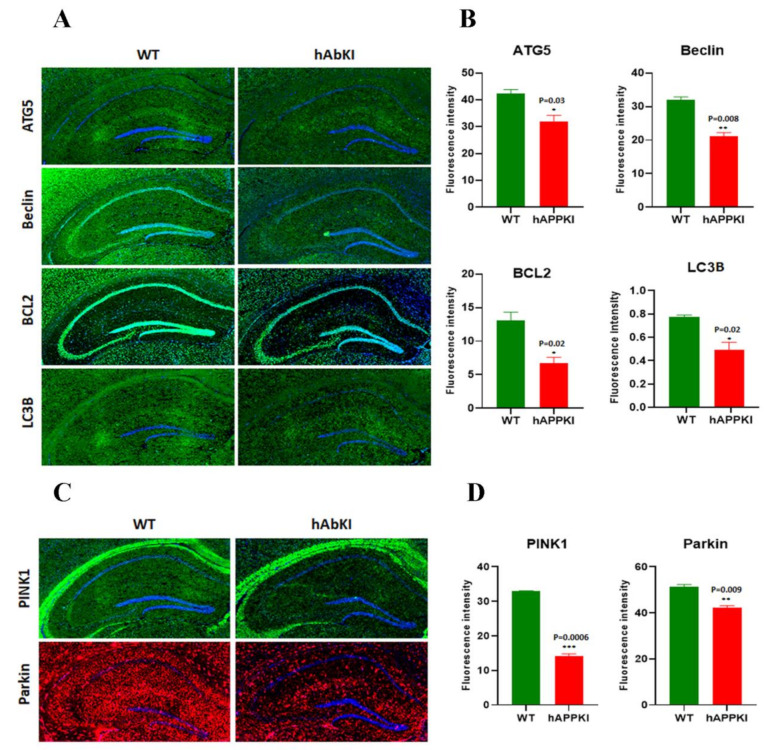
Immunofluorescence analysis of hippocampal mitochondrial-autophagy proteins and mitophagy proteins in seven-month-old WT and hAbKI mice. (**A**) Immunofluorescence staining and quantitative-immunofluorescence analysis of autophagy proteins in WT and hAbKI mice. (**B**) Autophagy proteins ATG5 (*p* = 0.03), Beclin (*p* = 0.008), BCL2 (*p* = 0.02) and LC3B (*p* = 0.02) were also reduced in hAbKI mice relative to WT mice. (**C**) Immunofluorescence staining and quantitative-immunofluorescence analysis of WT and hAbKI mice. (**D**) PINK1 and Parkin levels were significantly elevated (PINK1 *p* = 0.0006; Parkin *p* = 0.009) in the hAbKI mice. *, *p* = 0.01; **, *p* = 0.001; ***, *p* = 0.0001.

**Figure 7:**
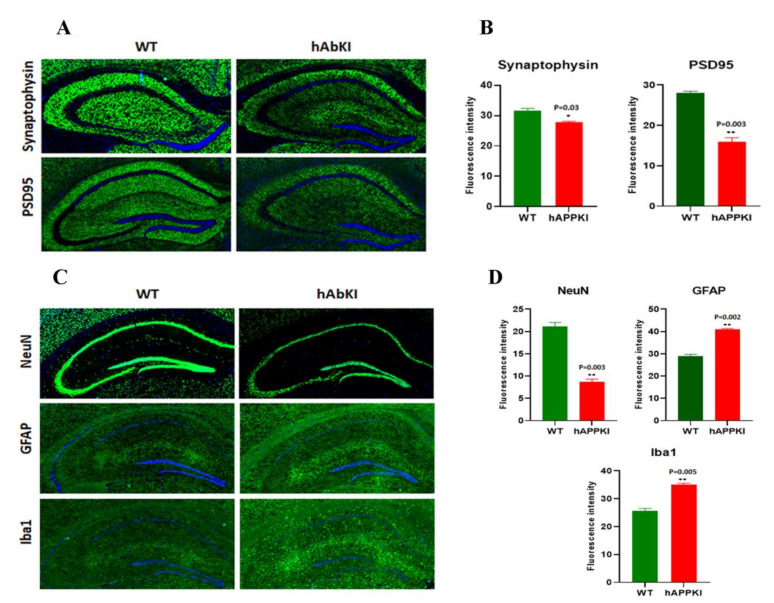
Immunofluorescence analysis of hippocampal synaptic proteins (synaptophysin and PSD95) and microglial, astrocytic, and neuronal proteins in seven-month-old WT and hAbKI mice. (**A**) Representative images of immunofluorescence and (**B**) quantitative-immunofluorescence analysis of synaptic proteins, synaptophysin and PSD95 in WT and hAbKI mice. (**C**) Representative images of immunofluorescence and (**D**) quantitative-immunofluorescence analysis of microglial Iba1 and astrocytic protein GFAP and neuronal protein NeuN in hAbKI mice relative to WT mice. *, *p* = 0.01; **, *p* = 0.001.

As shown in Figure 4A,B, mitochondrial-dynamic proteins were increased (Drp1, *p* = 0.0005; Fis1, *p* = 0.007) and fusion proteins were reduced (Mfn1, *p* = 0.001; Mfn2, *p* = 0.01; Opa1, *p* = 0.007) in hAbKI mice. Mitochondrial-biogenesis proteins were also reduced in hAbKI mice (Figure 5A,B); Autophagy proteins ATG5 (*p* = 0.03), Beclin (*p* = 0.008), BCL2 (*p* = 0.02) and LC3B (*p* = 0.02) were also reduced in hAbKI mice relative to WT mice (Figure 6A,B), and mitophagy proteins PINK1 (*p* = 0.0006) and Parkin (*p* = 0.009) were reduced (Figure 6C,D). Our immunofluorescence results on synaptic and dendritic proteins showed that synaptophysin (*p* = 0.03) and PDS95 (*p* = 0.003) were all reduced in seven-month-old hAbKI mice relative to WT mice (Figure 7A,B). Microglial marker Iba (*p* = 0.003) and astrocytic protein GFAP (*p* = 0.002) were significantly increased; on the other hand, neuronal protein NeuN was increased in hAbKI mice relative to WT mice (Figure 7C,D).

These observations strongly suggest that both the hippocampus and cerebral cortex were affected by human A-beta peptide in mice.

### 3.5. Dendritic Spine Density

We quantified dendritic length and number of spines per dendrite using Golgi–Cox staining in the hippocampus, cortex and dorsal raphe of seven-month-old hAbKI and WT mice in order to assess the effects of human A-beta on dendritic length and spines.

**Figure 8:**
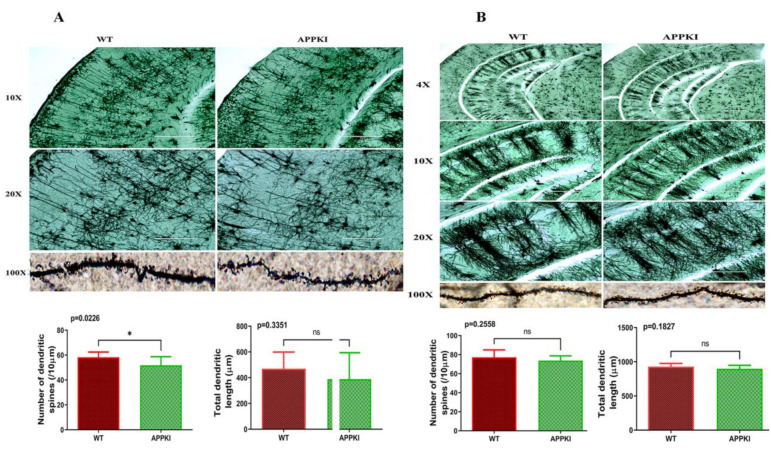
Golgi–Cox staining representing hippocampal dendritic-spine density in the brains of seven-month-old WT and hAbKI mice. (**A**) Represents Golgi–Cox staining at 4×, 10× and high magnification at 60×. (**B**) Represents quantification of spine density in the hAbKI and WT mice. Significantly reduced dendritic spines were found in hAbKI mice relative to WT mice. *, *p* = 0.01; ns, not significant.

Figure 8A shows representative images of dendrites from the cortices of seven-month-old hAbKI and WT mice. A significantly reduced number of dendritic spines was found in hAbKI mice (*p* = 0.0226) relative to WT mice. Dendritic length appeared to be reduced in hAbKI mice relative to WT mice, but the difference was not significant. 

Figure 8B shows representative images of hippocampal neurons from seven-month-old hAbKI and WT mice. The number of dendritic spines and dendritic length appeared to be reduced in hippocampal neurons in hAbKI mice relative to WT mice, but the changes were not significant.

### 3.6. Transmission Electron Microscopy

**Figure 9:**
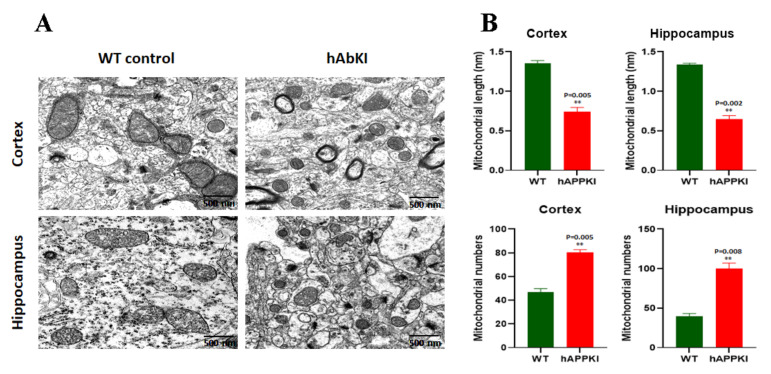
Transmission electron microscopy of cortical and hippocampal tissues from seven-month-old WT and hAbKI mice. Using transmission electron microscopy, we assessed mitochondrial number and length in cortical and hippocampal tissues from seven-month-old hAbKI and WT mice. (**A**) shows representative images of mitochondrial morphology in cortical and hippocampal areas of hAbKI and WT mice brains. (**B**) shows significantly increased mitochondrial number in the cortices (*p* = 0.005) and hippocampi (*p* = 0.008) of hAbKI mice relative to WT mice. Mitochondrial length is reduced in the cortices (*p* = 0.005) and hippocampi (*p* = 0.002) of hAbKI mice relative to WT mice. **, *p* = 0.001.

To determine the impact of human A-beta peptide on mitochondrial number and length, using transmission electron microscopy, we assessed the mitochondrial number and length in cortical and hippocampal tissues from seven-month-old hAbKI and WT mice. Figure 9B shows a significantly increased mitochondrial number in the cortices (*p* = 0.005) and hippocampi (*p* = 0.008) of hAbKI mice relative to WT mice. As expected, mitochondrial length is reduced in the cortices (*p* = 0.005) and hippocampi (*p* = 0.002) of hAbKI mice relative to WT mice.

### 3.7. Amyloid-Beta Levels

**Figure 10:**
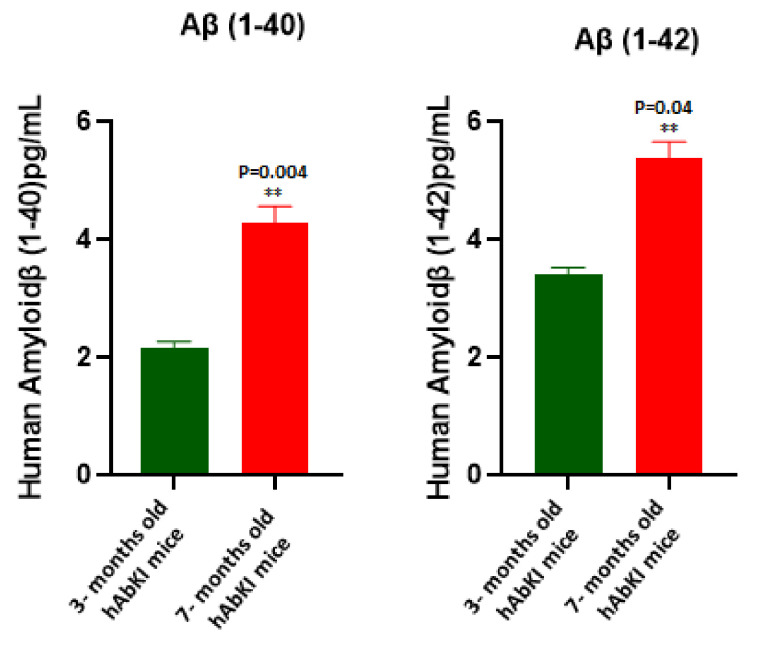
Amyloid-beta levels in three- and seven-month-old hAbKI mice. Using sandwich ELISA kit (IBL), we measured soluble Aβ1-40 and 1–42 levels in three- and seven-month-old homozygous hAbKI mice. Both Aβ1-40 and 1–42 levels were found in three months old mice and it progressively increased with age in seven-month-old mice. **, *p* = 0.001.

To understand the impact of human amyloid-beta peptide, using sandwich ELISA kit (IBL), we measured soluble Aβ1-40 and 1–42 levels in three- and seven-month-old homozygous hAbKI mice. As shown in Figure 10, we found both 1–40 (*p* = 0.04) and 1–42 levels (*p* = 0.004) in three-month-old mice and progressively increased in seven-month-old mice. These observations indicate the age-dependent increase of amyloid-beta in homozygous hAbKI mice and the involvement of impaired phenotypic behavior.

### 3.8. Mitochondrial Function

**Figure 11:**
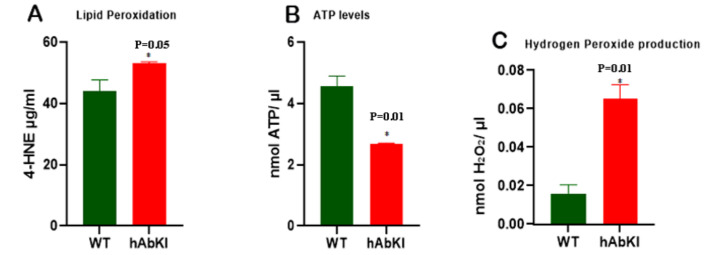
Mitochondrial function. Mitochondrial functional parameters, including lipid peroxi-dation (4-hydroxy-nonenol) (**A**), mitochondrial ATP (**B**) and hydrogen peroxide (**C**) were measured in the cortices of 7-month-old hAbKI and age-matched WT mice. Data are mean ± SD (*n* = 5 for each group). Significantly increased levels of 4-hydroxy-nonenol (lipid peroxidation), hydrogen peroxide and significantly decreased mitochondrial ATP levels were found in hAbKI mice relative to WT mice. * *p* = 0.01.

To understand the impact of human amyloid-beta on mitochondrial function, we assessed lipid peroxidation (4-hydro-nonenol), mitochondrial ATP and hydrogen peroxide (free radicals) in cortical tissues from seven-month-old hAbKI and age-matched WT mice. As shown in Figure 11, both lipid-peroxidation levels (*p* = 0.05) and hydrogen-peroxide production (*p* = 0.01) were significantly increased in hAbKI mice relative to WT mice. On the other hand, mitochondrial ATP levels (*p* = 0.01) were significantly decreased in hAbKI mice. These observations indicate that mitochondrial function is defective early on in the disease process.

## 4. Discussion

The purpose of our study was to investigate early cellular, molecular, morphological and pathological changes in homozygous humanized A-beta knock-in mice. Using seven-month-old homozygous hAbKI mice and age-matched WT control mice, we first studied behavioral phenotypes, including spatial learning and memory, locomotor activity, working memory and motor coordination. Using qRT-PCR, immunoblotting, and immunofluorescence analyses, we also assessed mRNA abundance and protein levels of mitochondrial-dynamic, mitochondrial-biogenesis, synaptic, mitophagy and autophagy genes; in addition, using transmission electron microscopy and Golgi–Cox staining, we assessed ultrastructural mitochondrial changes (length and number) and dendritic spines (length and number). We also assessed mitochondrial function by studying free radicals, mitochondrial ATP and lipid peroxidation.

Our extensive behavioral analyses revealed that seven-month-old hAbKI mice showed impairments in motor coordination (rotarod), reduced locomotor activity and exploration (open field), impairments in working memory (Y-maze) and spatial learning and memory (Morris Water Maze). Our analysis of mRNA levels revealed the increased expression of mitochondrial-fission genes and the reduced expression of mitochondrial-fusion, biogenesis, synaptic and autophagy and mitophagy genes in seven-month-old hAbKI mice. Our Western-blot analysis agreed with the observed mRNA changes in mitochondrial-, synaptic-, autophagy- and mitophagy-protein levels. Our transmission-electron-microscopy results revealed increased mitochondrial fragmentation and reduced mitochondrial length in both hippocampal and cortical tissues of seven-month-old hAbKI mice relative to WT mice.

Golgi–Cox staining revealed reduced dendritic spines in both the cerebral cortices and hippocampi of hAbKI mice, but statistical significance was observed only in the cortices. Dendritic length appeared to be reduced in hippocampal and cortical tissues, but the change was not significant. Mitochondrial function was defective, meaning an increased free-radical production, lipid peroxidation and reduced mitochondrial ATP in hAbKI mice. Together, these observations suggest that behavioral changes, changes in the expression of mitochondrial and synaptic genes, and ultrastructural changes in mitochondria are present early on in the disease progression of hAbKI mice.

To determine whether early behavioral impairments were present in hAbKI mice, we assessed locomotor activity/exploration abilities (open-field test), motor learning and coordination (rotarod), working memory (Y-maze) and spatial learning and memory (Morris Water Maze) in seven-month-old hAbKI mice and age-matched WT mice. These analyses were not performed in hAbKI mice by Frank LaFerla’s group in their initial characterization of the model. As mentioned in our results section, hAbKI showed reduced competence at treading on the rotarod (Figure 1A). These observations suggest that human A-beta peptide induced impairments in motor learning and coordination activities in hAbKI mice.

Humanized A-beta knock-in mice showed reduced locomotor and exploratory behaviors (Figure 1B). hAbKI mice also exhibited reduced spatial recognition and working memory, as evidenced by the Y-maze test (Figure 1D). The percentage of spontaneous alternation was significantly reduced in the hAbKI mice compared to WT mice. In the Morris-Water-Maze test, hAbKI mice took longer to find the platform. These data strongly suggest that human A-beta peptide is toxic and causes neuronal changes that impair motor learning and coordination, diminish exploratory and locomotor activities, and interfere with spatial-learning and memory functions. In a previous study of behavioral analysis in hAbKI mice, the authors found deficits in fear conditioning at 10 months and novel-object recognition at 14 months of age. Our current study found impairments in seven-month-old mice, indicating that human A-beta peptide is toxic and induces deficits in homozygous hAbKI mice much earlier than 10 months of age.

### 4.1. mRNA Abundance and Protein Levels

To determine if human A-beta peptide alters mRNA abundance and protein levels of mitochondrial-dynamic, biogenesis, synaptic, autophagy and mitophagy genes, we studied mRNA and protein levels in seven-month-old hAbKI mice and age-matched WT mice. As shown in the results section, both the mRNA and protein levels of mitochondrial-fission genes were increased, while mRNA and proteins levels of fusion, synaptic, autophagy and mitophagy genes were decreased in hAbKI mice relative to WT mice. Our current findings are consistent with observations from other AD mouse models, with the main difference being that the fold changes in mRNA expression were smaller in magnitude in hAbKI mice. The changes mRNA expressions were in agreement with the changes in protein levels for the genes studied. These observations strongly indicate that human A-beta peptide is toxic and alters mRNA and protein levels even at seven months of age. As discussed earlier [35,36], reduced mitochondrial biogenesis is an early feature of AD. We also found reduced mRNA and protein levels of autophagy and mitophagy genes in the hAbKI mice, indicating that human A-beta peptide alone induces defective autophagy and mitophagy activities in AD.

### 4.2. Spine Density Changes and Synaptic Activity

Mounting evidence indicates that dendritic spines are the true indicators of synaptic activity and cognitive function [9,18,30,37,38]. Therefore, intact and healthy dendritic spines are important to maintaining synaptic function. In AD, a reduced number of dendritic spines and reduced levels of synaptic proteins have been extensively reported in mouse models of AD [35,39] and in post-mortem brains from AD patients [37,40]. These observations prompted us to investigate the effects of human A-beta on dendritic spines and synaptic proteins, synaptophysin and PSD95 and dendritic protein MAP2 in seven-month-old hAbKI mice. As reported above, we observed reduced dendritic spines in the cerebral cortices of hAbKI mice relative to WT mice. In addition, in the current study, we found reduced levels of synaptic proteins, synaptophysin and PSD95 and dendritic protein MAP2 in seven-month-old hAbKI mice. Overall, these observations strongly suggest that that human A-beta induces synaptic toxicity, leading to reduced dendritic spines and likely cognitive and behavioral changes.

### 4.3. Mitochondrial Morphology Changes

Our electron-microscopy findings revealed a significantly increased number of mitochondria in hippocampal and cortical tissues of seven-month-old hAbKI mice relative to age-matched WT mice. The mitochondrial length was significantly decreased in both the hippocampus and cerebral cortical tissues of seven-month-old hAbKI mice relative to WT mice. These observations also agree with our earlier in vitro studies in which HT22 cells were transfected with an APP-overexpression plasmid [36,41] and earlier in vivo studies in using transgenic APP mouse models [35]. Overall, human A-beta peptide is toxic and fragments mitochondria in mice.

### 4.4. Human A-Beta Peptide Toxicity

Increasing evidence suggests that human Aβ peptide has different antagonistic biological characteristics. On one hand, it has trophic and even antioxidant properties; on the other hand, it has a high diversity of toxic properties [42]. Recent research on human and rodent Aβ peptides revealed that rodent amyloid-beta (roAβ) is identical to huAβ, except for three amino acids within the proposed heme-binding motif. Human Aβ binds heme tightly compared to roAβ and forms a peroxidase complex. While both roAβ and huAβ form aggregates equally, rodents lack the AD-like neuropathology and cognitive impairments [43]. In humans, a high huA-beta/heme ratio increases the peroxidase activity, leading to toxic properties and age-dependent synaptic and cognitive impairments [43]. Physiological expression and accumulation of human Aβ over time causes toxicity related to multiple cellular changes such as oxidative stress, mitochondrial diffusion, alterations in membrane permeability, inflammation, synaptic dysfunction, and excitotoxicity through its interaction with some neurotransmitter receptors and other mitochondrial proteins.

### 4.5. Mitochondrial Function

Our mitochondrial functional assays revealed increased free radicals and lipid peroxidation (4-hydroxy-nonenol) and reduced mitochondrial ATP in seven-month-old hAbKI mice relative to WT mice, indicating that mitochondrial function is defective at early stages of late-onset AD mice. These findings concur with our earlier studies of APP mice [35] and APP cell cultures [36,41], i.e., that human Aβ peptide induces free radicals and cause mitochondrial dysfunction early on in the disease process.

### 4.6. Oxidative Stress

The pro-oxidant effect shown by Aβ has been extensively studied using paramagnetic electronic resonance, a highly sensitive method for the direct detection of free radicals in the brain [42]. The Aβ peptide self-aggregates and interacts with a large number of proteins to induce free radicals and cause toxicity. Increasing evidence suggests that Aβ enters mitochondria where it induces free radicals and causes mitochondrial dysfunction [37,44]. Further, it is well established that the first 15 amino acids (histidines 6, 13, and 14 and the tyrosine in the 10th position) of Aβ have metal-binding sites that induce free radicals and oxidation in the brain [42]. Overall, these interactions cause oxidative damage in the brains of hAbKI mice.

### 4.7. Inflammation

As expected, inflammation is increased, as evidenced by data from Western-blot and immunofluorescence analyses of microglial marker Iba and astrocytic marker GFAP and the reduced neuronal marker NeuN.

## 5. Conclusions

In summary, we found motor coordination, locomotor-activity and spatial-learning and memory impairments in middle-aged (seven-months) hAbKI mice. Markers of inflammation, mitophagy, and autophagy were also affected, in addition to markers of mitochondrial dynamics, biogenesis and synaptic activity in hAbKI mice. Mitochondrial function was defective in hAbKI mice. Dendritic spines were affected in the cortex and hippocampus. Overall, our study utilized hAbKI mice of considerably younger ages than those used in prior studies, and morphological, cellular, and behavioral changes were detectable. This suggests that hAbKI mice might serve as a model for preclinical studies of preventive therapies.

## Figures and Tables

**Table 1:** Summary of q RT-PCR oligonucleotide primers used in measuring mRNA expression in mitochondrial structural, biogenesis, synaptic genes and autophagy and mitophagy genes.

Gene	DNA Sequence (5′-3′)	PCR Product Size
**Mitochondrial Structural Genes**		
Drp1	Forward Primer ATGCCAGCAAGTCCACAGAA	86
	Reverse Primer	
Fis1	Forward Primer CAAAGAGGAACAGCGGGACT	95
	Reverse Primer ACAGCCCTCGCACATACTTT	
Mfn1	Forward Primer GCAGACAGCACATGGAGAGA	83
	Reverse Primer GATCCGATTCCGAGCTTCCG	
Mfn2	Forward Primer TGCACCGCCATATAGAGGAAG	78
	Reverse Primer TCTGCAGTGAACTGGCAATG	
Opa1	Forward Primer ACCTTGCCAGTTTAGCTCCC	82
	Reverse Primer TTGGGACCTGCAGTGAAGAA	
**Mitochondrial Biogenesis Genes**		
PGC1α	Forward Primer GCAGTCGCAACATGCTCAAG	83
	Reverse Primer GGGAACCCTTGGGGTCATTT	
Nrf1	Forward Primer AGAAACGGAAACGGCTCAT	96
	Reverse Primer CATCCAACGTGGCTCTGAGT	
Nrf2	Forward Primer ATGGAGCAAGTTTGGCAGGA	96
	Reverse Primer GCTGGGAACAGAGGTAGTAT	
TFAM	Forward Primer TCCACAGAACAGCTACCCAA	84
	Reverse Primer CCACAGGGCTGCAATTTTCC	
**Synaptic genes**		
Synaptophysin	Forward Primer CTGCGTTAAAGGGGGCACTA	81
	Reverse Primer ACAGCCACGGTGACAAAGAA	
PSD95	Forward Primer CTTCATCCTTGCTGGGGGTC	90
	Reverse Primer TTGCGGAGGTCAACACCATT	
Synapsin 1	Forward Primer TGAGGACATCAGTGTCGGGTAA	64
	Reverse Primer GGCAATCTGCTCAAGCATAGC	
Synapsin 2	Forward Primer TCCCACTCATTGAGCAGACATACT	
	Reverse Primer GGGAACGTAGGAAGCGTAAGC	
Synaptobrevin 1	Forward Primer TGCTGCCAAGCTAAAAAGGAA	68
	Reverse Primer CAGATAGCTCCCAGCATGATCA	
Synaptobrevin 2	Forward Primer GGGACCAGAAGTTGTCGGAG	89
	Reverse Primer CTTGAGCTTGGCTGCACTTG	
Neurogranin	Forward Primer CTCCAAGCCAGACGACGATA	83
	Reverse Primer AACTCGCCTGGATTTTGGCT	
**Mitophagy genes**		
PINK1	Forward Primer CCATCGGGATCTCAAGTCCG	70
	Reverse Primer GATCACTAGCCAGGGACAGC	
Parkin	Forward Primer AGAGGTCCAGTTAAACCCACC	90
	Reverse Primer GAGGGTTGCTTGTTTGCAGG	
**Autophagy genes**		
ATG5	Forward Primer TCCATCCAAGGATGCGGTTG	95
	Reverse Primer TCTGCATTTCGTTGATCACTTGAC	
BCL2	Forward Primer TCCTTCCAGCCTGAGAGCAA	73
	Reverse Primer GCCTGAGAGGAGACGTCCTG	
LC3B	Forward Primer TCCACTCCCATCTCCGAAGT	94
	Reverse Primer TTGCTGTCCCGAATGTCTCC	
Beta-actin	Forward Primer AGAAGCTGTGCTATGTTGCTCTA	91
	Reverse Primer TCAGGCAGCTCATAGCTCTTC	

**Table 2:** Summary of antibody dilutions and conditions used in the immunoblotting analysis of mitochondrial dynamics, mitochondrial biogenesis, synaptic mitophagy and autophagy proteins in mitophagy-enhancer-treated and untreated hAbKI mice.

Marker Primary Antibody—Species and Dilution	Purchased from Company, City and State	Secondary Antibody, Dilution	Purchased from Company, City and State
Drp1 Rabbit polyclonal 1:500	Novus Biological, Littleton, CO	Donkey anti-rabbit HRP 1:10 000	GE Healthcare Amersham, Piscataway, NJ
Fis1 Rabbit polyclonal 1:500	Protein Tech Group, Inc., Chicago, IL	Donkey anti-rabbit HRP 1:10 000	GE Healthcare Amersham, Piscataway, NJ
Mfn1 Rabbit polyclonal 1:400	Abcam, Cambridge, MA	Donkey anti-rabbit HRP 1:10 000	GE Healthcare Amersham, Piscataway, NJ
Mfn2 Rabbit polyclonal 1:400	Abcam, Cambridge, MA	Donkey anti-rabbit HRP 1:10 000	GE Healthcare Amersham, Piscataway, NJ
OPA1 Rabbit polyclonal 1:500	Novus Biological, Littleton, CO	Donkey anti-rabbit HRP 1:10 000	GE Healthcare Amersham, Piscataway, NJ
SYN Rabbit monoclonal 1:400	Abcam, Cambridge, MA	Donkey anti-rabbit HRP 1:10 000	GE Healthcare Amersham, Piscataway, NJ
PGC1a Rabbit polyclonal 1:500	Novus Biological, Littleton, CO	Donkey anti-rabbit HRP 1:10 000	GE Healthcare Amersham, Piscataway, NJ
NRF1 Rabbit polyclonal 1:300	Novus Biological, Littleton, CO	Donkey anti-rabbit HRP 1:10 000	GE Healthcare Amersham, Piscataway, NJ
NRF2 Rabbit polyclonal 1:300	Novus Biological, Littleton, CO	Donkey anti-rabbit HRP 1:10 000	GE Healthcare Amersham, Piscataway, NJ
TFAM Rabbit polyclonal 1:300	Novus Biological, Littleton, CO	Donkey anti-rabbit HRP 1:10 000	GE Healthcare Amersham, Piscataway, NJ
PINK1 Rabbit polyclonal 1:500	Novus Biological, Littleton, CO	Donkey anti-rabbit HRP 1:10 000	GE Healthcare Amersham, Piscataway, NJ
Parkin Mouse polyclonal 1:500	Novus Biological, Littleton, CO	Sheep anti-mouse HRP 1:10 000	GE Healthcare Amersham, Piscataway, NJ
ATG5 Rabbit Polyclonal 1: 1000 dilutions	Novus Biological, Littleton, CO	Donkey Anti-rabbit HRP 1:10,000	GE Healthcare Amersham, Piscataway, NJ
LC3B Rabbit Polyclonal 1: 1000 dilutions	Novus Biological, Littleton, CO	Donkey Anti-rabbit HRP 1:10,000	GE Healthcare Amersham, Piscataway, NJ
Beclin-1 Rabbit Polyclonal 1: 1000 dilutions	Novus Biological, Littleton, CO	Donkey Anti-rabbit HRP 1:10,000	GE Healthcare Amersham, Piscataway, NJ
Bcl-2 Rabbit Polyclonal 1: 1000 dilutions	Novus Biological, Littleton, CO	Donkey Anti-rabbit HRP 1:10,000	GE Healthcare Amersham, Piscataway, NJ
Iba1/AIF-1 2 Rabbit monoclonal 1: 1000 dilutions	Cell Signaling Technology, Inc., Danvers, MA	Donkey Anti-rabbit HRP 1:10,000	GE Healthcare Amersham, Piscataway, NJ
Anti-NeuN Rabbit monoclonal 1: 1000 dilutions	Abcam, Cambridge, MA	Donkey Anti-rabbit HRP 1:10,000	GE Healthcare Amersham, Piscataway, NJ
B-Actin Mouse Monoclonal 1:2000	Millipore Sigma (Burlington, MA, USA)	Sheep anti-mouse HRP 1:10,000	GE Healthcare Amersham, Piscataway, NJ

**Table 3:** Summary of antibody dilutions and conditions used in the immunofluorescence analysis of mitochondrial dynamics, mitochondrial biogenesis, synaptic mitophagy and autophagy proteins in mitophagy-enhancer-treated and untreated hAbKI mice.

Marker Primary Antibody—Species and Dilution	Purchased from Company, City and State	Secondary Antibody, Dilution	Purchased from Company, City and State
Drp1 Rabbit polyclonal 1:100	Novus Biological, Littleton, CO	Donkey anti-rabbit HRP 1:200	Invitrogen, Waltham, MA
Fis1 Rabbit polyclonal 1:100	Protein Tech Group, Inc., Chicago, IL	Donkey anti-rabbit HRP 1:200	GE Healthcare Amersham, Piscataway, NJ
Mfn1 Rabbit polyclonal 1:100	Abcam, Cambridge, MA	Donkey anti-rabbit HRP 1:200	GE Healthcare Amersham, Piscataway, NJ
Mfn2 Rabbit polyclonal 1:100	Abcam, Cambridge, MA	Donkey anti-rabbit HRP 1:200	GE Healthcare Amersham, Piscataway, NJ
OPA1 Rabbit polyclonal 1:100	Novus Biological, Littleton, CO	Donkey anti-rabbit HRP 1:200	GE Healthcare Amersham, Piscataway, NJ
SYN Rabbit monoclonal 1:400	Abcam, Cambridge, MA	Donkey anti-rabbit HRP 1:200	GE Healthcare Amersham, Piscataway, NJ
PGC1a Rabbit polyclonal 1:100	Novus Biological, Littleton, CO	Donkey anti-rabbit HRP 1:200	GE Healthcare Amersham, Piscataway, NJ
NRF1 Rabbit polyclonal 1:100	Novus Biological, Littleton, CO	Donkey anti-rabbit HRP 1:200	GE Healthcare Amersham, Piscataway, NJ
NRF2 Rabbit polyclonal 1:100	Novus Biological, Littleton, CO	Donkey anti-rabbit HRP 1:200	GE Healthcare Amersham, Piscataway, NJ
TFAM Rabbit polyclonal 1:100	Novus Biological, Littleton, CO	Donkey anti-rabbit HRP 1:200	GE Healthcare Amersham, Piscataway, NJ
PINK1 Rabbit polyclonal 1:100	Novus Biological, Littleton, CO	Donkey anti-rabbit HRP 1:200	GE Healthcare Amersham, Piscataway, NJ
Parkin Mouse polyclonal 1:100	Novus Biological, Littleton, CO	Sheep anti-mouse HRP 1:200	GE Healthcare Amersham, Piscataway, NJ
ATG5 Rabbit Polyclonal 1: 100 dilutions	Novus Biological, Littleton, CO	Donkey Anti-rabbit HRP 1:200	GE Healthcare Amersham, Piscataway, NJ
LC3B Rabbit Polyclonal 1: 100 dilutions	Novus Biological, Littleton, CO	Donkey Anti-rabbit HRP 1:200	GE Healthcare Amersham, Piscataway, NJ
Beclin-1 Rabbit Polyclonal 1: 100 dilutions	Novus Biological, Littleton, CO	Donkey Anti-rabbit HRP 1:200	GE Healthcare Amersham, Piscataway, NJ
Bcl-2 Rabbit Polyclonal 1: 100 dilutions	Novus Biological, Littleton, CO	Donkey Anti-rabbit HRP 1:200	GE Healthcare Amersham, Piscataway, NJ
Iba1/AIF-1 2 Rabbit monoclonal 1: 100 dilutions	Cell Signaling Technology, Inc., Danvers, MA	Donkey Anti-rabbit HRP 1:200	GE Healthcare Amersham, Piscataway, NJ
Anti-NeuN Rabbit monoclonal 1: 100 dilutions	Abcam, Cambridge, MA	Donkey Anti-rabbit HRP 1:200	GE Healthcare Amersham, Piscataway, NJ

**Table 4:** Fold changes of mRNA expression in mitochondrial-dynamic-, biogenesis-, synaptic-, autophagy- and mitophagy-related genes in hAbKI mice in comparison with WT mice.

	Genes	mRNA Fold Change in AAPKI Mice
Mitochondrial genes	Drp1	1.8 ***
	Fis1	1.69 **
	Mfn1	−2 **
	Mfn2	−1.33 *
	OPA1	−1.38
Biogenesis genes	PGC1 alpha	−1.7 *
	Nrf1	−1.4
	Nrf2	−1.36
	TFAM	−2 *
Synaptic genes	Synaptophysin	−2.04 **
	PSD95	−1.1 *
	Snap25	−2.27 **
	Synapsin 1	−1.4 *
	Synapsin 2	−5 ***
	Synaptobrevin 1	−1.26
	Synaptobrevin 2	−2.4 *
	Neurogranin	−1.54 *
Mitophagy genes	PINK1	−2 **
	Parkin	−1.16
Autophagy genes	ATG5	−1.6 **
	BCL2	−1.25 *
	LC3B	−1.33

*, *p* = 0.01; **, *p* = 0.001; ***, *p* = 0.0001.

## Data Availability

Current work is on characterization of mouse model and all the details are avaialable in our article.
